# Direct observation of solid-state reversed transformation from crystals to quasicrystals in a Mg alloy

**DOI:** 10.1038/srep09816

**Published:** 2015-06-12

**Authors:** Jian-Fang Liu, Zhi-Qing Yang, Heng-Qiang Ye

**Affiliations:** 1 Shenyang National Laboratory for Materials Science, Institute of Metal Research, Chinese Academy of Sciences, Shenyang 110016, China

## Abstract

**Phase transformation of quasicrystals is of interest in various fields of science and technology. Interestingly, we directly observed unexpected solid-state epitaxial nucleation and growth of Zn**_**6**_**Mg**_**3**_**Y icosahedral quasicrystals in a Mg alloy at about 573 K which is about 300 K below the melting point of Zn**_**6**_**Mg**_**3**_**Y, in contrast to formation of quasicrystals through solidification that was usually found in many alloys. Maximizing local packing density of atoms associated with segregation of Y and Zn in Mg adjacent to Mg/Zn**_**3**_**MgY interfaces triggered atomic rearrangement in Mg to form icosahedra coupled epitaxially with surface distorted icosahedra of Zn**_**3**_**MgY, which plays a critical role in the nucleation of icosahedral clusters. A local Zn:Mg:Y ratio close to 6:3:1, corresponding to a valence electron concentration of about 2.15, should have been reached to trigger the formation of quasicrystals at Mg/Zn**_**3**_**MgY interfaces. The solid-state icosahedral ordering in crystals opens a new window for growing quasicrystals and understanding their atomic origin mechanisms. Epitaxial growth of quasicrystals onto crystals can modify the surface/interface structures and properties of crystalline materials.**

The discovery of quasicrystals fundamentally alters how scientists in chemistry, physics and materials science conceive of solid matters^1^,^2^,^3^,^4^,^5^,^6^,^7^,^8^. Since the discovery of quasicrystals in melt-spun ribbons of an Al-Mn alloy^1^, quasiperiodic ordering states have been found in numerous intermetallic alloys^9^,^10^,^11^,^12^,^13^, soft materials^4^,^5^,^6^,^7^,^14^, oxide films^15^ and natural materials^16^,^17^. Extensive studies have provided explicit knowledge for understanding and modeling the atomic arrangements in intermetallic quasicrystals, as well as their origin mechanisms^18^,^19^,^20^. Mackay, Bergman or Cd-Yb icosahedral clusters are building blocks of three-dimensional (3D) icosahedral quasicrystals (IQC)^9^,^10^,^11^,^21^. Intermetallic quasicrystals are usually formed in undercooled liquids^1^,^11^,^12^ or metallic glasses^13^ both containing icosahedral atomic clusters^13^,^22^,^23^. Quasicrystals could also be formed through a peritectic reaction during cooling^24^. Computer simulations showed that a dodecagonal quasicrystal nucleus grew through assimilation of icosahedral clusters in a supercooled liquid^25^.

Besides comprehensive studies on the formation of quasicrystals, transformation from quasicrystals to crystals have also been investigated^13^,^26^,^27^,^28^,^29^. Thermally induced transformations from quasicrystals to their approximants have been found in various systems^26^, such as IQCs of Al-Cu-Fe, Ti-Zr-Ni and Mg-Zn-Al, decagonal quasicrystals of Al-Co-Ni and Al-Pd, as well as octagonal quasicrystals of Cr-Ni-Si and Mn-Si-Al. Precipitation of quasicrystalline particles and their crystalline approximants of R-phase during isothermal heating at respectively 748 K and 823 K, as well as their reversible transformation through minor rearrangements of atoms, were observed in a maraging steel^29^. Interestingly, Abe and Tsai^27^ reported that a hexagonal phase in a Zn_65_Mg_25_Y_10_ alloy transformed into quasicrystals upon heating at 873 K which is around the melting point of the alloy^30^. But, it remains a challenge to realize solid-state crystal-to-quasicrystal transformation at relatively low temperatures when the movement and assimilation of whole (icosahedral) atomic clusters are less likely, and entropy does not predominate the free energy, especially in crystalline metallic systems without crystalline approximants.

In this paper, we elucidate the mechanism of solid-state nucleation and growth of Zn_6_Mg_3_Y IQC at interfaces between H phase Zn_3_MgY crystal and hexagonal Mg matrix in a Mg-4.3Zn-0.7Y (at.%) alloy by *in situ* heating transmission electron microscopy (TEM). The H phase Zn_3_MgY has a hexagonal crystal structure (a = 0.91 nm, c = 0.94 nm), and it is not an approximant^31^. IQC nanoparticles started to form at Zn_3_MgY/Mg interfaces upon heating up to about 573 K which is almost 300 K below the melting point of the Zn_6_Mg_3_Y quasicrystal phase^30^. The newly formed IQC nanoparticles have specific orientation relationships with the parent H phase, providing important clues to advance atomic-level understanding of the solid-state origin of 3D IQC in metallic systems.

## Results

### *In situ* observations on the solid-state formation of quasicrystals

**Figure 1:**
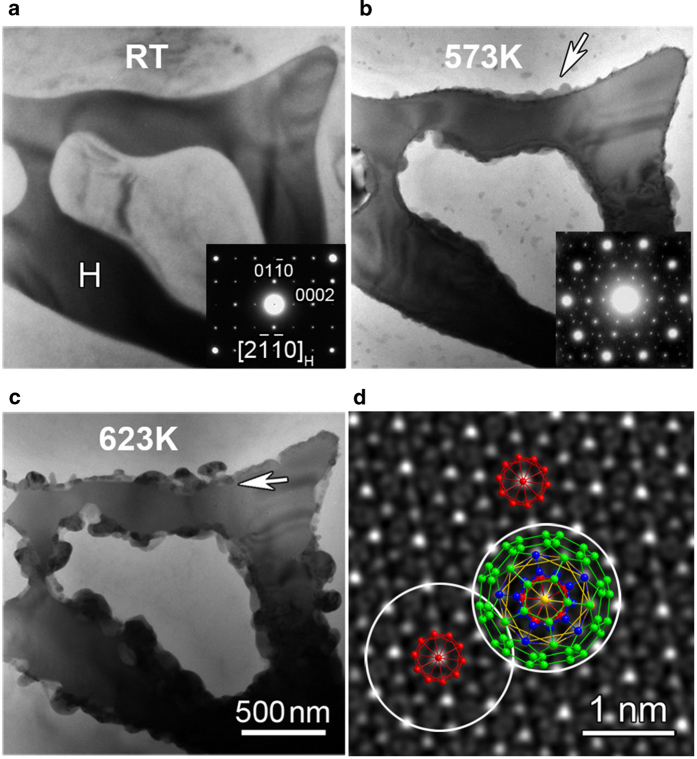
**Sequential *in situ* heating TEM images at different temperatures showing the transformation from H to IQC phases. a**, RT. **b**, 573 K, 15 min. **c**, 623 K, 9 min. **d**, Five-fold zone axis high-resolution Z-contrast image of an IQC particle. The insets in (**a**) and (**b**) are [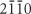]_H_ and 5-fold axis SAED patterns of Zn_3_MgY and IQC phases, respectively. The arrows in (**b**) and (**c**) indicate IQC particles grown into Mg and Zn_3_MgY, respectively. In (**d**), three core icosahedra are highlighted by red dots; white circles outline icosahedral clusters ~2 nm in diameter, showing overlapping growth; a 5-fold axis projection of one hierarchical icosahedral cluster up to its fourth fullerene-structure layer is overlapped on the image.

Figure 1a shows Zn_3_MgY crystals in Mg matrix and the corresponding [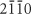] zone selected area electric diffraction (SAED) pattern. Zn_3_MgY/Mg interfaces are smooth and clean, without any other particles in the sample at room temperature. Raising the temperature to 573 K, small particles started to appear at Zn_3_MgY/Mg interfaces and grew into Mg, while there was no obvious change in the H phase, as indicated by the arrow in Fig. 1b. These particles grew up with increasing the temperature (Fig. 1c). These newly formed particles were identified to be Zn_6_Mg_3_Y IQC by SAED analysis (inset in Fig. 1b) and high-resolution Z-contrast imaging (Fig. 1d). Bergman icosahedral clusters are the building units of Zn_6_Mg_3_Y IQC^9^,^18^,^19^,^20^,^21^. Core icosahedra with atomic distances of about 0.30 nm in the IQC phase are indicated by red dots, while blue, yellow and green dots and lines outline respectively the second to the fourth layers of a hierarchical Bergman cluster (Fig. 1d). The atomic projections along the five-fold axis of the Bergman icosahedral cluster match well with positions of intensity peaks in the high-resolution Z-contrast image, as shown in Fig. 1d. Figure S1 demonstrates good agreement between simulated and experimental high-resolution Z-contrast images along both five-fold and two-fold axes. It is also found that local size of the Zn_3_MgY particle kept on decreasing due to growth of IQC particles, indicating that growth of IQC is at the expense of Zn_3_MgY. Since the formation of IQC particles was initiated on the side of Mg matrix (Fig. 1b), a layer of Mg enriched with Zn and Y should appear first as a result of diffusion, in order to trigger the nucleation of IQC. The Zn_3_MgY particle can be the source of Y. The diffusing Y atoms in Mg could not leave far away from the Zn_3_MgY/Mg interface due to their relatively low diffusivity, resulting in local Y segregation in Mg adjacent to the interface; strong attraction between Zn and Y could drive diffusion of Zn atoms from surrounding matrix toward the interface^28^, leading to the nucleation of IQC at certain Zn and Y concentrations ultimately.

Interestingly, diffraction contrast of H-phase particle, Mg grain and the newly formed IQC particles was always present during the whole transformation process (Fig. 1b,c), demonstrating that all phases were not melted. This rules out the possibility of peritectic reaction^24^ for the observed formation of IQC. The previously reported transformation from crystals to IQCs^27^ at about 873 K is probably a solidification process^32^, since the temperature is quite close to the melting point of the material^30^. The present crystal-to-quasicrystal transformation was started at about 573 K which is about 300 K below the melting point of Zn_6_Mg_3_Y quasicrystals, providing firm evidence for the solid-state origin of quasicrystals in crystalline materials. The solid-state nucleation and growth of IQCs are principally different from the appearance of small IQC particles through breaking and dissolution of larger ones^33^. Furthermore, the formation of quasicrystals at Zn_3_MgY/Mg interface could not be realized through minor rearrangement of atoms like that happened during reversible transformation between quasicrystals and their approximants of R phase in a maraging steel^29^, since Zn_3_MgY is not an approximant^31^.

### Orientation relationships and atomic structure of interfaces

**Figure 2:**
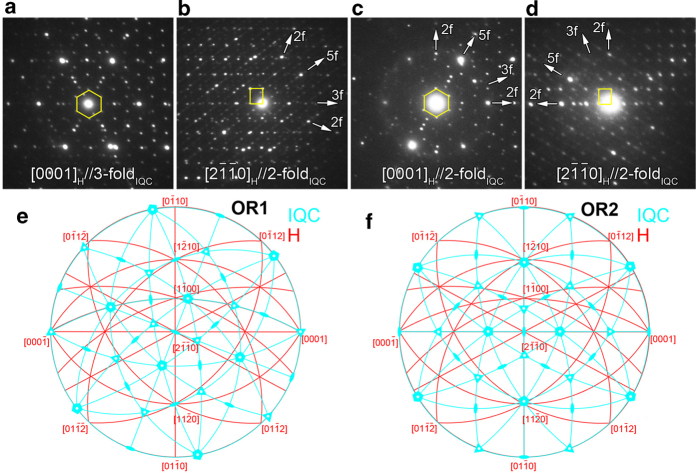
**Orientation relationships between IQC and H phases. a**-**d**, Composite SAED patterns of H and IQC phases. **e**,**f**, Stereograms showing the two specific orientation relationships between H and IQC phases. No exact superposition happens between the 5-fold axis of IQC and either [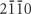]_H_ or [0001]_H_ in both (**e**) and (**f**).

Figures 2a–d show respectively two groups of composite SAED patterns for two different IQC particles formed at Zn_3_MgY/Mg interfaces, demonstrating specific orientation relationships (ORs) between the newly formed Zn_6_Mg_3_Y IQC quasicrystals and Zn_3_MgY crystals. Two specific ORs (referred as OR1 and OR2, respectively) between H and IQC phases are determined based on SAED and corresponding stereogram analyses (Figs. 2e,f). The composite SAED patterns shown in Figs. 2a and b represent orientation relationship OR1 (Fig. 2e), which is described as 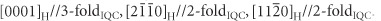. The composite SAED patterns shown in Figs. 2c and d represent orientation relationship OR2 (Fig. 2f), which is 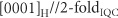, 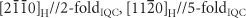 (there is a small misorientation of ~1.7°). OR1- and OR2-oriented IQC particles coexist on the same Zn_3_MgY grain. But, the newly formed IQC particles have no apparent orientation relationship with Mg matrix (Fig. S2). Therefore, the epitaxial relationships suggest that nucleation and growth of IQC particles have intimate relation with the H phase, from a crystallographic point of view.

**Figure 3:**
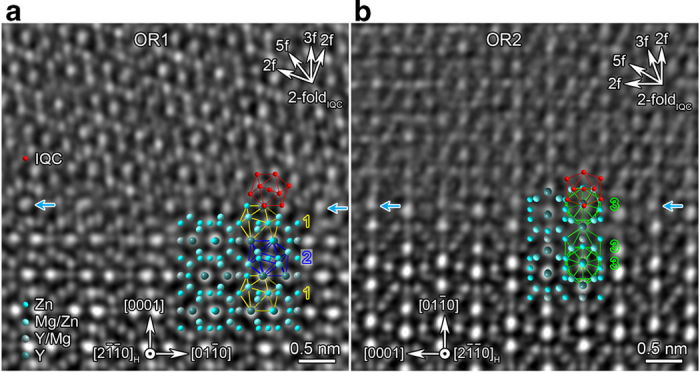
**High-resolution STEM observations on H/IQC interface structures. a**, OR1. **b**, OR2. One icosahedron of the IQC is indicated in red; projections for icosahedra Zn1, Zn2 and Zn3 in the H phase are respectively outlined by yellow, blue and green lines, and numbered by 1–3 correspondingly. Those brightest intensity peaks represent atomic columns composed of Y and Zn in the interior of H phase. The decreased intensities for those atomic columns (indicated by blue arrows) of H phase are due to interdiffusion of Y and Mg. Red dots show a core icosahedron projected along a 2-fold axis. The central red dot represents atoms from other shells.

In the unit cell of Zn_3_MgY, there are 28 distorted icosahedral atomic sites among all 36 atomic sites^31^. These atoms form four kinds of icosahedral coordination, denoted as Zn1, Zn2, Zn3 (marked by yellow, blue and green lines in Fig. 3) and Mg/Zn icosahedra. Neighboring distorted icosahedra in Zn_3_MgY interconnect with each other through sharing a vertex, an edge, a face, or a pentagonal bipyramid (interpenetrated) (Fig. S3). To understand the formation mechanism of IQC particles on the atomic level, IQC/Zn_3_MgY interfaces were characterized by high-angle annular dark-field scanning transmission electron microscopy (HAADF-STEM). Figure 3a shows the interface between an OR1-oriented IQC particle and one Zn_3_MgY grain. The interface is basically parallel to (0001)_H_ plane. The 2-fold axis of icosahedra Zn1 and Zn2 is along the 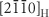 direction. At the interface, an icosahedron marked by red in the IQC phase is connected with an icosahedron Zn1 marked by yellow lines in the H phase through sharing a vertex, which is the same connectivity between icosahedra Zn2 and Zn1 in Zn_3_MgY crystal. An atomic resolution HAADF-STEM image and a 4-layer Bergman icosahedral cluster along its 2-fold axis are shown in Fig. S4.

Figure 3b shows an IQC particle that has orientation relationship OR2 with H phase. The interface is parallel to the prismatic 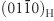 plane. Icosahedra Zn3 with their 5-fold or 2-fold axes parallel to the 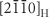 direction are outlined by green lines. Two neighbouring icosahedra with respectively their 5-fold or 2-fold axes parallel to the 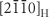 form an interpenetrated pair in H phase, as shown in Fig. 3b. An icosahedron marked by red lines in the IQC phase also forms a interpenetrated pair with an icosahedron Zn3 marked by green lines in the H phase at the interface, inheriting the connectivity between Zn3 icosahedra in H phase. For both interfaces shown in Fig. 3, the red icosahedra in the IQC are connected with the surface icosahedra of Zn_3_MgY, forming pairs with the same connectivity as those inside Zn_3_MgY. In other words, the new icosahedra of IQC were epitaxially grown on surfaces of H phase.

**Figure 4:**
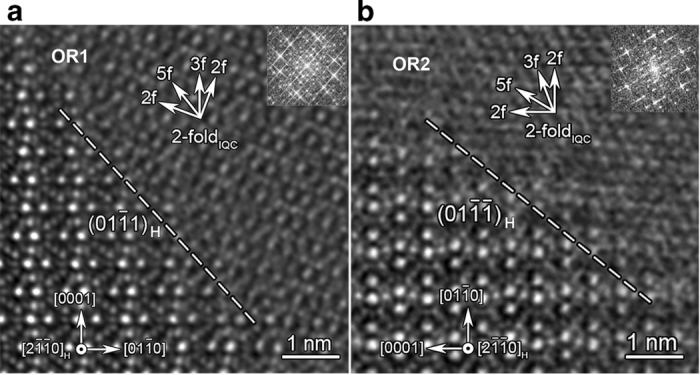
**High-resolution STEM images on H/IQC interface structures. a**, OR1-oriented IQC on 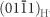. **b**, OR2-oriented IQC on 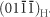.

OR1-oriented and OR2-oriented IQC particles were also observed on interfaces terminated at lattice planes other than (0001)_H_ and 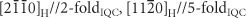, as shown in Fig. 4 and Fig. S5.

**Figure 5:**
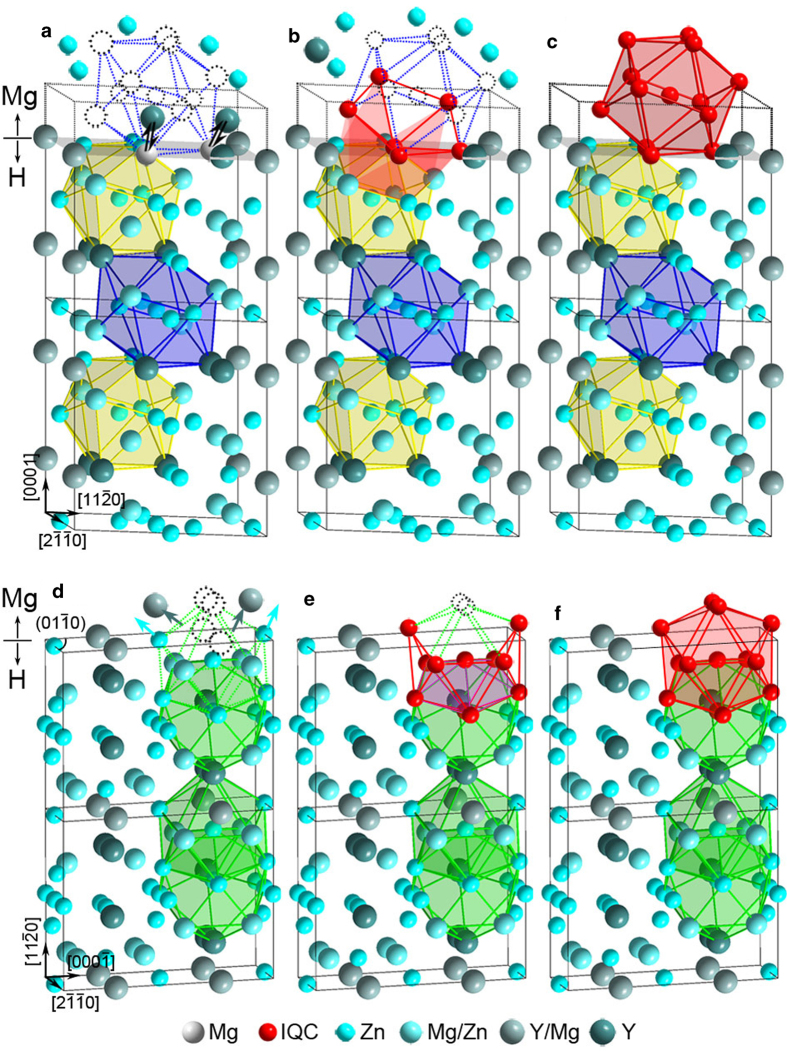
**Schematic illustrations showing formation of new icosahedra on the surface of H phase through tetrahedrally packing atoms onto distorted icosahedra of the H phase. a**-**c**, The new icosahedron has OR1 with the H phase, was connected with the distorted icosahedra by sharing a vertex. Two surface Y atoms of Zn_3_MgY were replaced by Mg as a result of interdiffusion in (**a**). **d**-**f**, The new icosahedron has OR2 with the H phase, was connected with the distorted icosahedra by sharing a pentagonal bipyramid as indicated by shaded area in (**e**). Open dotted circles indicate lattice positions for the red icosahedron. Hexagonal Mg atoms are not shown for a purpose of clarity.

In solid crystal lattice, it is principally impossible to move and assimilate a whole atomic icosahedral cluster to other structures, like that happened in liquids^24^. Instead, it is more energetically and geometrically feasible to form new structures through diffusion and rearrangement of atoms^13^,^26^. In order to form a hierarchical icosahedral cluster, a core icosahedron should be formed first. Figures 5a–c are proposed schematic illustrations showing the formation of an icosahedron in Mg through tetrahedrally packing atoms at the (0001)_H_−terminated surface. As depicted in Fig. 5a, Y and Zn segregation in a layer of Mg should first occur in the immediate vicinity of the Mg/Zn_3_MgY interface. At some point with proper local concentration of Zn and Y, nucleation of new structures should take place through relocation of atoms from their original Mg lattice sites to proper positions, driven by the decrease in free energy. The atoms would be incorporated gradually into tetrahedral positions indicated by dotted circles belonging to an icosahedron (Fig. 5b), since icosahedral ordering can minimize specific volume and local energy^34^. Further relocation of Zn, Y and Mg atoms could lead to the formation of a whole hierarchical icosahedral cluster. It is most likely that surfaces of Zn_3_MgY would act as templates for the rearrangements of atoms in the immediately adjacent Mg lattice, so as to form an icosahedron epitaxially onto the crystalline Zn_3_MgY particle through sharing a vertices with a surface icosahedron Zn1 of Zn_3_MgY (Fig. 5c), in order to minimize the interfacial energy. At the same time of atom relocation in Mg solid-solution, relaxation of distorted icosahedra and atom rearrangement should occur within the Y-depleted, but Mg-enriched surface layer of H phase, which transforms the corresponding layer into IQC. Similar atomic processes to form icosahedra can occur at 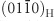
−terminated interfaces, where the newly formed icosahedron form an interpenetrated pair with one in H phase, as shown in Figs. 5d–f. Therefore, epitaxial formation of an icosahedron on surfaces of H phase is quite similar to the growth of H phase itself, if we neglect the change in composition and lattice relaxation.

**Figure 6:**
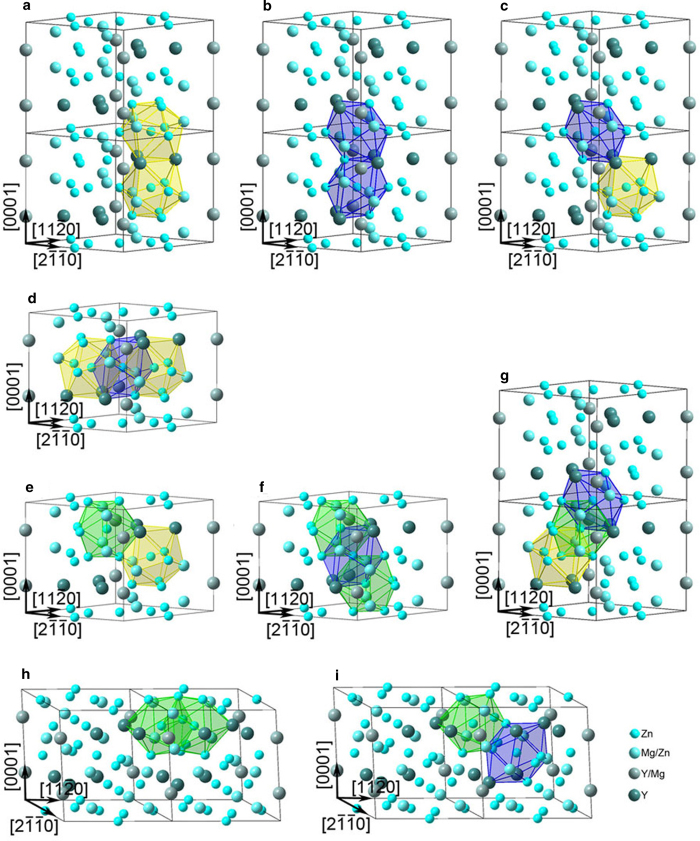
**Correlation between the interconnection of neighboring icosahedra in the H phase and IQC/H orientation relationships.** **a**-**g**, OR1. **h**,**i**, OR2. The icosahedra share triangular faces (**a**,**b**,**e**,**i**), edge (**c**), vertex (**d**,**f**,**g**), and pentagonal bipyramids (**d**,**f**,**g**,**h**).

Good epitaxy can be seen at all IQC/Zn_3_MgY interfaces (Figs. 3 and 4, Fig. S5). This can be reasonably attributed to the variety of epitaxial connectivity between icosahedra in 3D space, as shown in Fig. 6. Therefore, it is highly possible to form nanoparticles of IQC epitaxially on the whole surfaces of H phase particles. Unfortunately, IQC and H phases could not be imaged with atomic-resolution simultaneously along 5-fold axis of IQC, due to intrinsic misorientation between 5-fold_IQC_ and [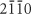]_H_ (Figs. 2 and S6).

OR1-oriented IQC particles (Figs. 3a,4a, S5) could nucleate and grow on Zn_3_MgY surfaces terminated at basal, prismatic or pyramid planes, if interfacial icosahedral pairs were formed through inheriting connectivity shown in Figs. 6a–c, Fig. 6d or Figs. 6e–g, respectively. OR2-oriented IQC particles would form on Zn_3_MgY surfaces terminated at prismatic (Fig. 3b) or pyramid (Fig. 4b) planes of H-phase if epitaxial icosahedra were nucleated through forming pairs as those shown in Figs. 6h or i, respectively. The two specific orientation relationships between IQC and H phases can thus be attributed to the epitaxial formation of icosahedra at surfaces of H phase, which entails minimum interfacial energy^35^. It can be expected that larger Bergman icosahedral clusters, and ultimately IQC particles will be produced, given that a local Zn:Mg:Y ratio close to 6:3:1 was retained with continued interdiffusion (Fig. 1).

## Discussion

Valence electron concentration (e/a) of compounds is an important factor dominating their formation of quasicrystals^9^,^36^. Quasicrystal formation is thus sensitive to the composition of compounds, since e/a of compounds is directly determined by their composition. Only tiny fluctuation of e/a is permitted in order to form quasicrystals in alloys, which corresponds to a quite narrow composition region. In order to form Zn-Mg-Y IQCs, e/a should be in the range of 2.0–2.15^9^,^36^. The e/a of Zn_6_Mg_3_Y IQC is 2.1. The e/a of H phase Zn_3_MgY is 2.2 which is larger than the upper limit of 2.15 for quasicrystals in this material system^36^, so it is a crystal, instead of a quasicrystal.

Dense packing of spherical particles have been widely used to model the structure of liquids and solids^37^,^38^,^39^,^40^. An icosahedron consists of 20 slightly distorted tetrahedra sharing a common vertex at its center, representing a “best” packing cluster of atoms with low-energy state and high stability^13^,^23^,^38^,^40^. The presence of icosahedral clusters has been demonstrated in both metallic liquids and glasses^23^,^41^. Mixtures of atoms with different sizes may form more complex structures with denser packing of atoms than cubic and hexagonal close sphere packings^26^. The atomic radii of Zn, Mg and Y are 0.137 nm, 0.160 nm and 0.181 nm, respectively. The packing density of atoms in Bergman icosahedral clusters in Zn_6_Mg_3_Y IQC is about respectively 27% and 56% higher than the hexagonal Zn_3_MgY and Mg crystals, according to their atomic structures and lattice parameters. There is plenty of room to obtain the densest atomic packings with lower free energy in both Mg solid solution and Y-depleted Zn_3_MgY phase, driving the crystal-to-quasicrystal transformation. The tetrahedrally close-packed structures formed by interconnected icosahedra occur frequently in intermetallic compounds. IQC phase can also be described as tetrahedrally close-packed structure for its hierarchical feature of icosahedral clusters^9^,^10^,^11^,^18^,^34^,^42^. Whether these energetically favorable clusters are packed periodically or quasiperiodically depends on the following factors: their shape and the relationship between their chemical composition and that of the whole phase in the case of intermetallic quasicrystals^26^.

A Zn:Mg:Y ratio close to 6:3:1 should be achieved within immediate neighborhood of the interface during the solid-state nucleation and growth of IQC particles. Rearrangement of atoms in the hexagonal Mg lattice at the interface was activated by forming epitaxial icosahedra on the crystalline surfaces of Zn_3_MgY (Figs. 3, 4, 5), leading to denser packing of atoms locally. The nucleated icosahedral clusters are energetically stable due to their proper valence electron concentration^9^,^36^, as well as the local dense packing^23^,^34^ and low-energy epitaxial IQC/H interface^35^. Besides the effect of valence electron concentration, replacing some larger Y atoms by smaller Mg atoms in Zn_3_MgY influences the crystal-to-quasicrystal transformation in another two aspects: (i) modifying the distortion of icosahedra, (ii) inducing slight shuffle of other non-icosahedral atoms in Zn_3_MgY to join icosahedral clusters. Therefore, minimization of specific local volume and energy drives the gradual relocation of atoms from their original crystal lattice positions in the parent phases to icosahedral lattice positions, leading to icosahedral clusters (~2 nm in diameter) with the densest packing of atoms^34^ and valence electron concentration quite close to 2.15^9^,^36^. This is a critical step for the solid-state nucleation and growth of IQC at Zn_3_MgY/Mg interfaces. Those 2 nm icosahedral clusters follow the overlapping rule to grow into IQC particles^42^, as shown in Fig. 1d.

Mg-Zn-RE alloys with IQC strengthening phases are attractive lightweight engineering materials, since they show excellent mechanical performances at both ambient and elevated temperatures^43^. The special atomic structure and bonding at interfaces between IQC and Mg matrix lattice are believed playing an important role in the superior mechanical performances of this type of Mg alloys. However, Zn_3_MgRE (H phase) and Zn_3_Mg_3_RE_2_ (W phase) impair the ductility in IQC-strengthened Mg-Zn-RE alloys^44^. Moreover, IQC phases could be transformed into deleterious H phase and W phase during processing^45^. It is expected that the mechanical properties of the Mg-Zn-RE alloys would be improved if those H phase and W phase particles could be transformed to IQCs. Therefore, studies on solid-state crystal-to-quasicrystal transformations are of engineering importance^29^,^46^,^47^. Our *in situ* observations showed that the starting temperature for the formation of IQC particles at Zn_3_MgY/Mg interfaces was about 573 K which is almost 100 K below the phase transition temperature of Zn_6_Mg_3_Y IQC in Mg-Zn-Y alloys^28^. It is thus expected that no transformation of IQC to other deleterious crystalline phases will happen upon annealing such IQC-strengthened Mg alloys at the temperature range of 573–623 K. Our results may provide useful information to evoke new processing methods to optimize mechanical properties of this kind of Mg alloys through controlling transformation of deleterious crystals to IQCs.

In summary, we have shown solid-state formation of IQC phase at the expense of Zn_3_MgY crystals in a Mg-Zn-Y alloy at a temperature about 300 K below the IQC’s melting point. IQC particles were epitaxially nucleated and grown at surfaces of Zn_3_MgY. Surface icosahedra of Zn_3_MgY crystals acted as templates for triggering epitaxial nucleation of new icosahedra, and thus decreasing the nucleation barrier. The decrease in free energy associated with local icosahedral ordering with the densest packing of atoms and proper valence electron concentration plays an important role in the formation of IQC in crystalline systems. Low-energy state of the epitaxial interfaces is another key factor for the growth of quasicrystal particles. The occurrence of solid-state nucleation and growth of quasicrystals through rearrangement of individual atoms in a crystal system at low temperatures is in stark contrast to the assimilation of whole icosahedra in supercooled liquids, opening another window for understanding the origin and growth mechanisms of quasicrystals. Solid-state formation of quasicrystals at relatively low temperatures may have implications in structural and functional modification of crystalline materials.

## Methods

### Material and sample preparation

A Mg-Zn-Y ternary alloy with the nominal composition of Mg-4.3Zn-0.7Y (at.%) was produced from high purity Mg, Zn metals and a Mg-Y master alloy by high frequency induction under an argon atmosphere. Thin foil samples for electron microscopy observations were prepared by standard precision ion milling.

### Electron microscopy studies

*In situ* heating TEM experiments were processed with a GATAN 652 double tilt heating holder on a Tecnai G^2^ F20 TEM operated at 200 kV, equipped with a HAADF detector and X-ray energy dispersive spectroscopy systems. The *in situ* process is depicted in Fig. S7. The temperature was held for a period at each temperature to find any changes in interesting areas. At temperatures below 550 K, no structural changes were observed at the H/Mg interfaces in our samples even after heating the sample for one hour. At 573 K, IQC nanoparticles started to appear at the H/Mg interfaces after heating for about 3 minutes (inset in Fig. S6). To accelerate the growth, the temperature was increased to 603 and 623 K. Some primary high-resolution TEM observations were performed before and after the *in situ* experiments. Atomic-resolution HAADF-STEM characterizations of the samples were performed using a Titan G^2^ 60-300 electron microscope operated at 300 kV, equipped with double aberration correctors. HAADF-STEM images can provide information about the chemistries of each atomic column^18^,^20^.

## Additional Information

**How to cite this article**: Liu, J.-F. *et al.* Direct observation of solid-state reversed transformation from crystals to quasicrystals in a Mg alloy. *Sci. Rep.* **5**, 9816; doi: 10.1038/srep09816 (2015).

## Supplementary Material

Supplementary Information
