# Supplementary material for: Direct observation of solid-state reversed transformation from crystals to quasicrystals in a Mg alloy
Source: Sci Rep. 2015 Jun 12;5:9816. doi: 10.1038/srep09816 (PMC4464364; doi:10.1038/srep09816)
Supplement: Supplementary Information [file srep09816-s1.pdf]

Direct observation of solid-state reversed transformation from crystals to quasicrystals in a Mg alloy

Jian-Fang Liu, Zhi-Qing Yang, Heng-Qiang Ye

Correspondence: yangzq@imr.ac.cn

This supplementary information includes:

Supplementary Figures S1-S8.

Reference

1. Orientation relationships between H, IQC and Mg phases

In the as-cast alloy, the H phase has no apparent orientation relationship with the Mg matrix, through extensive electron diffraction analyses. High-resolution STEM observations also confirmed this, as shown in Fig. S2a. After the in situ heating process, the newly formed IQC particles have specific orientation relationships with the H phase, but both of them have not apparent orientation relationships with the Mg matrix (Fig. S2b).

2. Interconnection of distorted icosahedra in the H phase

In the structure of H phase, four independent atomic sites - three Zn sites and the mixed Mg/Zn site - have icosahedral coordination, denoted as Zn1, Zn2, Zn3 and Mg/Zn icosahedra (Supplementary Fig. 3)[1]. Due to the difference of the radii of Mg, Zn and Y atoms, these four kinds of icosahedra are distorted. With only two bigger Y atoms on its vertices, the Zn3 icosahedron is the least distorted one among them. On the contrary, the Zn2 icosahedron, has four Y atoms on its vertices, is the most distorted one. Similarly, the Mg/Zn icosahedron has three Y and one Mg/Y atoms on its vertices, so it is distorted. The four icosahedra are mutually interpenetrated by sharing a tetrahedron consisting of Zn1, Zn2, Zn3 and Mg/Zn vertices. Therefore, there are many interconnection types between the binary icosahedra, sharing vertices, triangular faces, edges and pentagonal bipyramids (interpenetrated), as shown in Figs. 5 and 6 of the manuscript.

3. Composition of H and IQC phases

Nano-beam energy-dispersive X-ray (EDX) spectroscopy measurements were performed to evaluate the chemical composition of both H phase crystals and IQC particles. The H and IQC phases in the Mg-4.3Zn-0.7Y(at.%) alloy have composition of $Mg_{24.4}Zn_{57.1}Y_{18.5}$ and $Mg_{32.2}Zn_{55.8}Y_{12}$, respectively. So, the H and IQC phase are simply described as Zn_3MgY and Zn_6Mg_3Y, respectively, in the paper. It is noted that such simplification does not have significant effect on the calculation of valence electron concentration of both compounds. Figure S8 shows composition change across an H/IQC interface between a Zn_3MgY crystal and a small Zn_6Mg_3Y quasicrystal particle obtained by a line-scan EDX chemical measurement, demonstrating the lower concentration of Y in the IQC particle, and slight concentration gradient of Mg at the interfacial region. Additionally, the EDX counts are obviously higher, and the counts for both Zn and Y are underestimated for the small IQC particle, since it is embedded in Mg matrix.

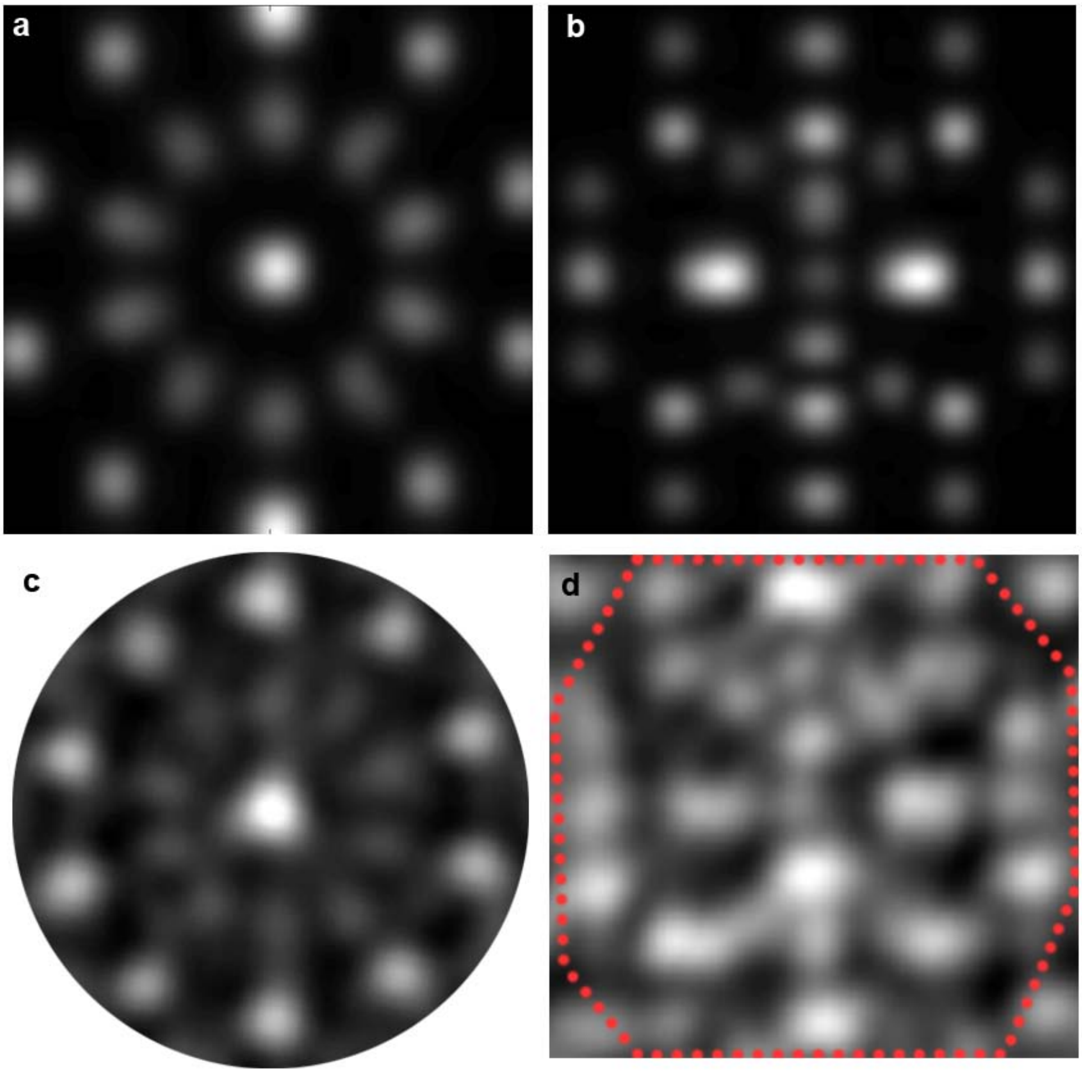

Figure S1. a, **b**, Simulated Z-contrast images along the 5-fold and 2-fold axes of a small icosahedral cluster, respectively. **c**, **d**, Experimental Z-contrast images recorded along the 5-fold and 2-fold axes of IQC, respectively. The dotted lines in **d** outline the region corresponding to the cluster used in simulating image shown in **b**. Good agreement between experimental and simulated images can be seen for both directions. The atomic models of the icosahedral cluster are shown in Figure 1**d** in the manuscript and Figure S4.

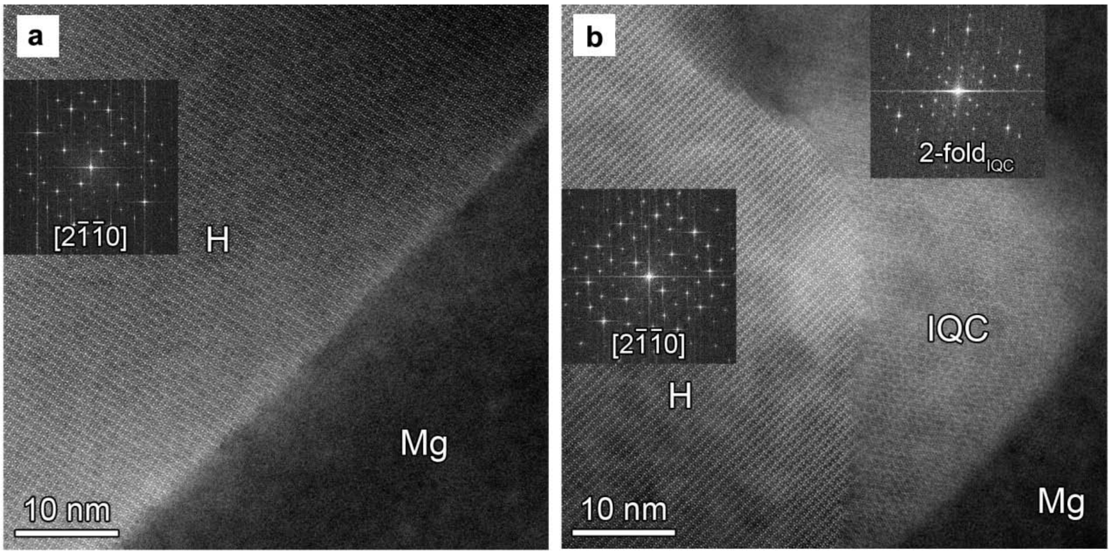

Figure S2. High-resolution HAADF-STEM observations on the interfaces before (**a**) and after (**b**) in-situ heating process. Both H-phase and IQC have no specific orientation relationship, according to the missing of lattice fringes in the Mg matrix. The inserts are fast Fourier transform images of corresponding areas.

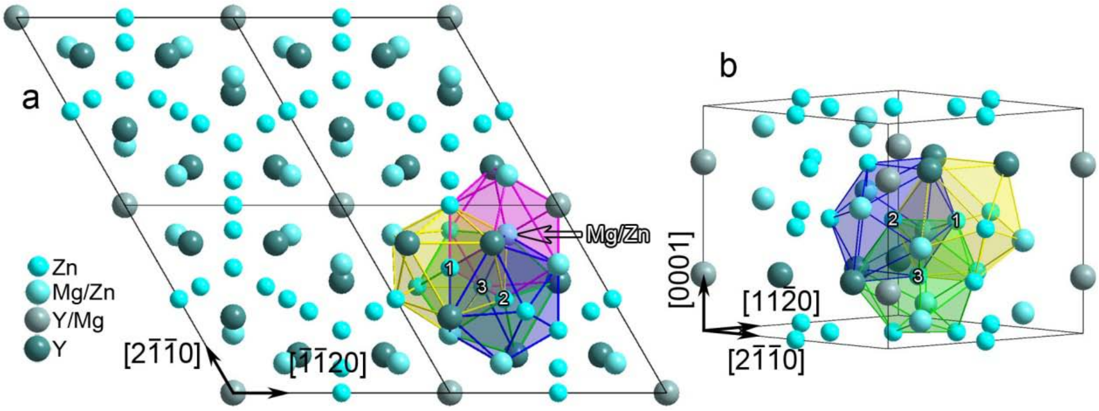

Figure S3. a, The [0001] projection, **b**, the 3D schematic illustration of the structure of the H phase. The Zn1, Zn2, Zn3 and Mg/Zn icosahedra are mutually interconnected by sharing a tetrahedron consisting of Zn1, Zn2, Zn3 and Mg/Zn vertices.

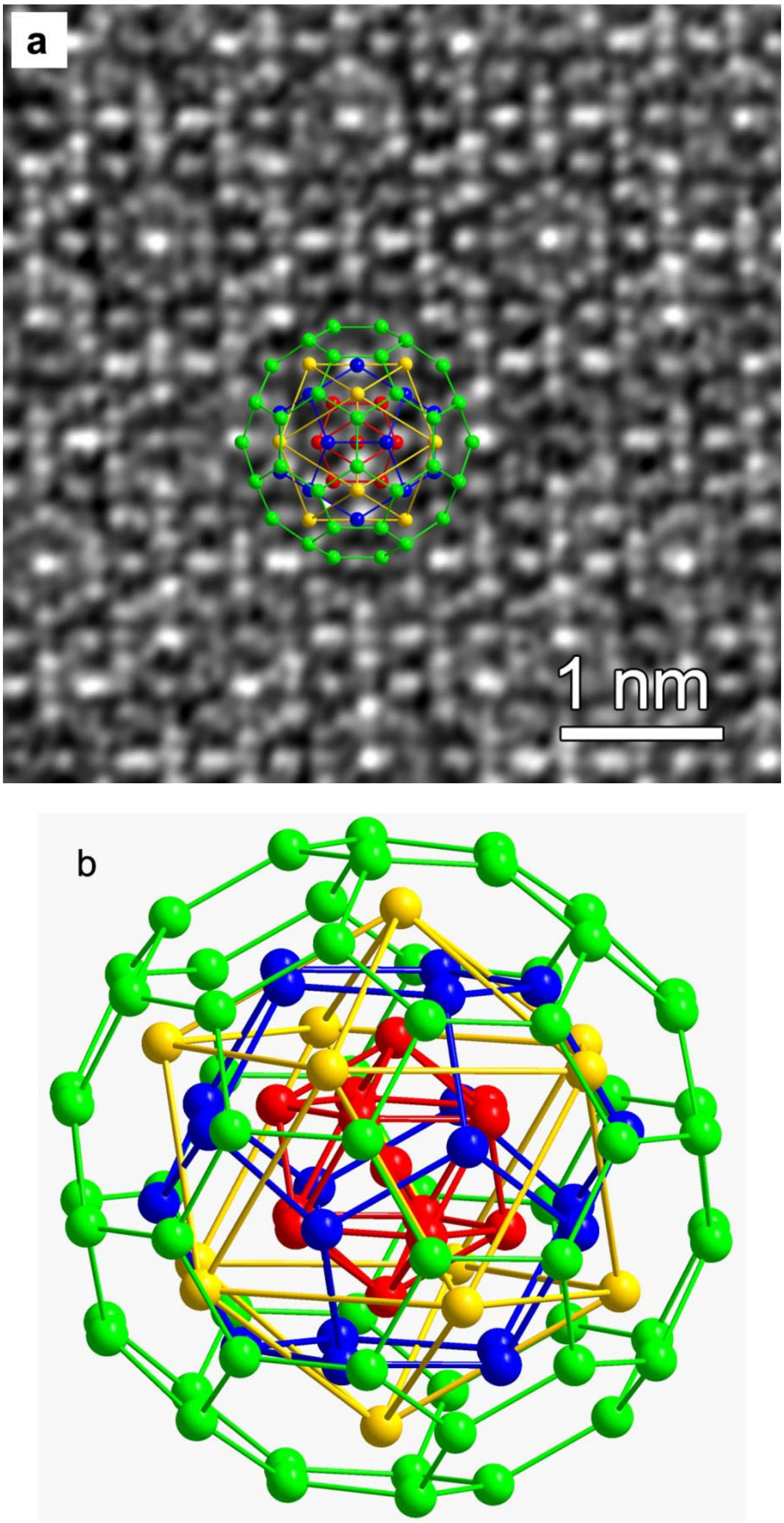

Figure S4. a, high-resolution HAADF-STEM image recorded along a 2-fold with a 4-layer icosahedral cluster, **b**, 3D view of the 4-layer icosahedral cluster shown in **a**.

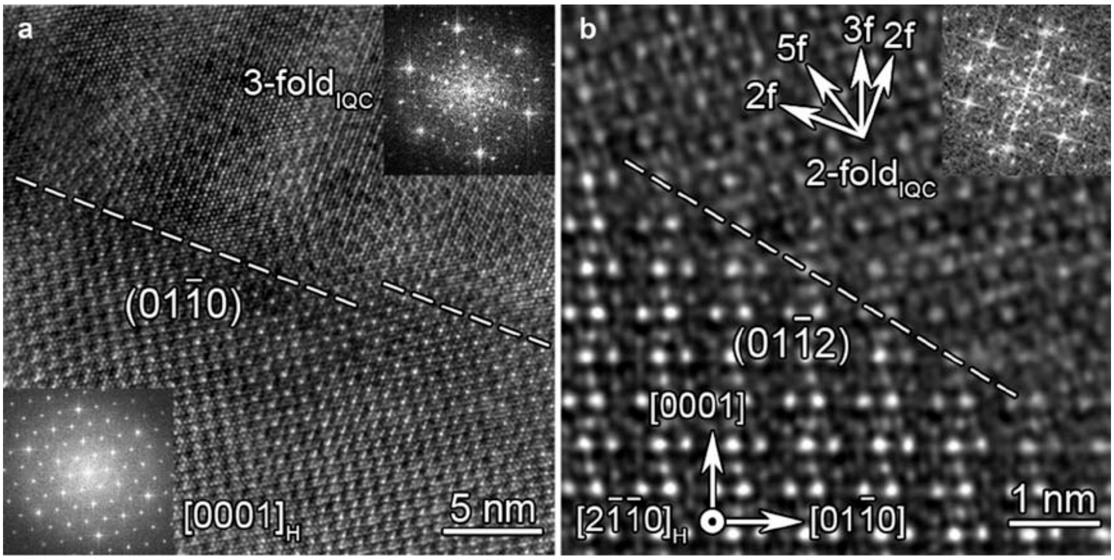

Figure S5. High-resolution TEM (**a**) and STEM (**b**) images showing OR1-orientated IQC particles formed on prismatic and pyramid planes of H phase.

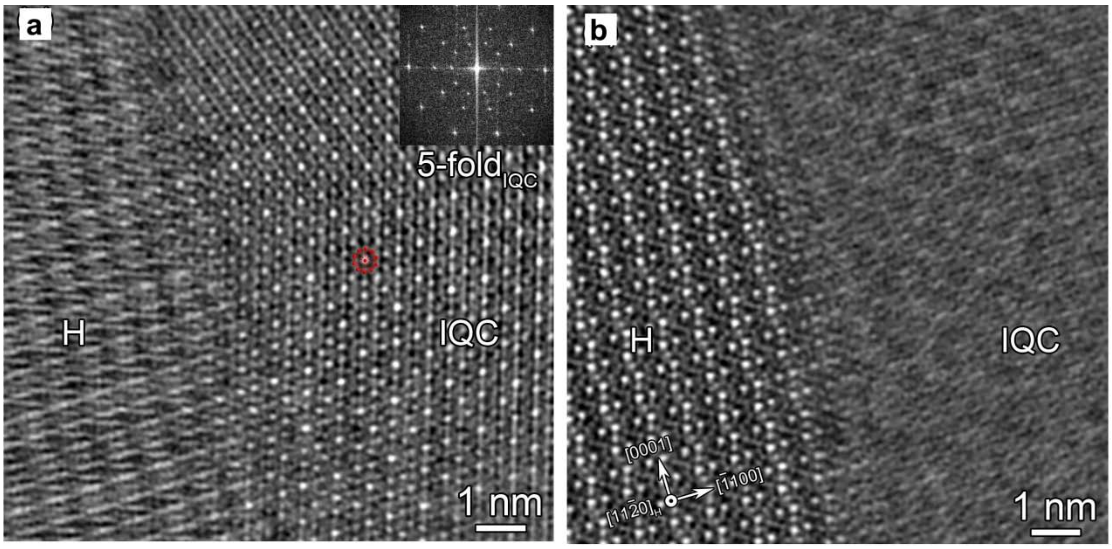

Figure S6. High-resolution STEM images recorded along (**a**) a five-fold axis of an IQC particle, (**b**) $[2\bar{1}\bar{1}0]_H$ axis for the same region with an OR2-oriented IQC particle.

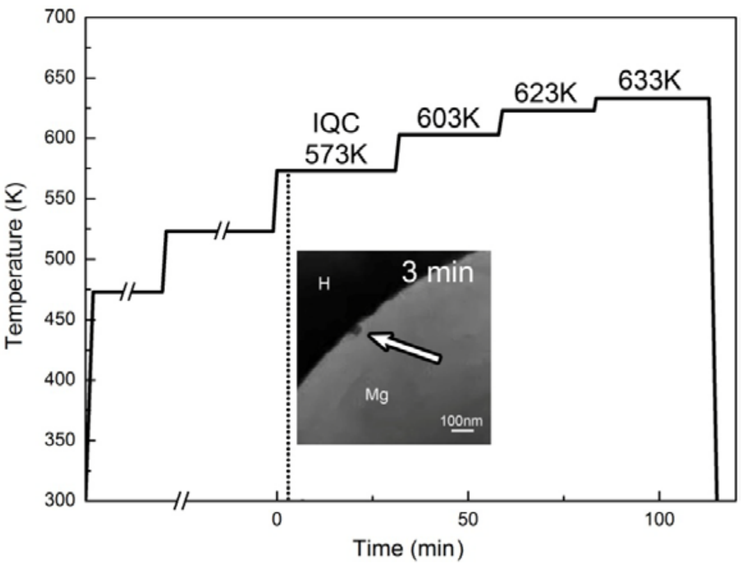

Figure S7. Schematic drawing of in situ heating process. The inset is a TEM image showing the formation of an IQC nanoparticle in Mg at the H/Mg interface after heating at 573 for about 3 minutes.

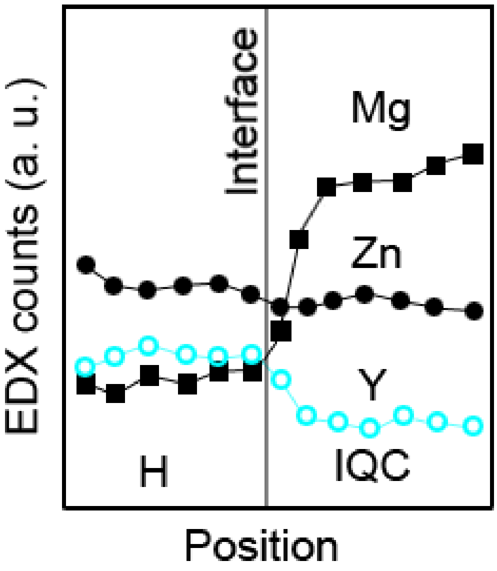

Figure S8. EDX line-scan showing composition variation across the H/IQC interface.

Reference

1. D. W. Deng, K. H. Kuo, Z. P. Luo, D. J. Miller, M. J. Kramer, and K. W. Dennis, Journal of Alloys and Compounds **373**, 156 (2004).
